# Systemic Amyloidosis: Lessons from β_2_-Microglobulin*

**DOI:** 10.1074/jbc.R115.639799

**Published:** 2015-03-06

**Authors:** Monica Stoppini, Vittorio Bellotti

**Affiliations:** From the ‡ Department of Molecular Medicine, Institute of Biochemistry, University of Pavia, 27100 Pavia, Italy and; the § Wolfson Drug Discovery Unit, Centre for Amyloidosis and Acute Phase Proteins, Division of Medicine, University College London, London NW3 2PF, United Kingdom

**Keywords:** Amyloid, Atomic Force Microscopy (AFM), Protein Aggregation, Protein Misfolding, Protein Structure, β2-Microglobulin, Fibrillogenesis in Vitro, Genetic Variant Asp76Asn, Mechanism of Amyloidogenesis

## Abstract

β_2_-Microglobulin is responsible for systemic amyloidosis affecting patients undergoing long-term hemodialysis. Its genetic variant D76N causes a very rare form of familial systemic amyloidosis. These two types of amyloidoses differ significantly in terms of the tissue localization of deposits and for major pathological features. Considering how the amyloidogenesis of the β_2_-microglobulin mechanism has been scrutinized in depth for the last three decades, the comparative analysis of molecular and pathological properties of wild type β_2_-microglobulin and of the D76N variant offers a unique opportunity to critically reconsider the current understanding of the relation between the protein's structural properties and its pathologic behavior.

## Introduction

In 1984, George Glenner and Caine Wong (1) provided the first chemical evidence that Alzheimer disease amyloid plaques were constituted by β-amyloid protein. The following year, the *Biochem. Biophys. Res. Commun.* study by the Fumitake Gejyo group (2) followed shortly after by the *J. Clin. Invest.* study by the Peter Gorevic and Blas Frangione team (3) both showed that the constituent of amyloid deposits of patients treated with chronic hemodialysis was the protein β_2_-microglobulin (β_2_-m).^2^ The further demonstration that the formation of amyloid required a persistently high plasma concentration of β_2_-m (4) was a clear proof that a critical concentration of a protein precursor is required for priming the formation of amyloid fibrils. Hence, the early biochemical characterization clearly showed that full-length non-mutated β_2_-m was abundantly present in natural amyloid fibrils (5). Further biochemical studies were carried out by Reynold Linke *et al.* (6) on different types of tissues, which included specimens of the carpal tunnel, as well as specimens derived from bone fractures caused by amyloid deposits and even urinary stones. From these studies emerged that in all natural amyloid deposits, the truncated species of β_2_-m lacking the six N-terminal residues was significantly represented (7). No other major post-translational modifications are apparently present in natural fibrillar β_2_-m. In amyloid deposits, the presence of the protein precursor's fragments is quite common. The truncation of extensive portions of the constant region is common in amyloidogenic light chains. Natural fibrils of apolipoprotein A-I mainly contain the N-terminal polypeptide corresponding to the first 100 residues, and the presence of transthyretin (TTR) fragments can be considered almost a hallmark of the cardiac involvement in TTR amyloidosis (8). The biochemical characterization of β_2_-m natural amyloid fibrils highlighted the co-deposition of other macromolecules. Some of them, such as serum amyloid P component (SAP) and glycosaminoglycans (GAGs), are generic co-constituents of all types of systemic amyloidosis (9, 10), but a few are apparently specifically associated with the β_2_-m-related form. In an *ante litteram* proteomic study, Campistol *et al.* (11, 12) showed that several anti-proteases are co-deposited in β_2_-m natural fibrils and that the presence of α_2_-macroglobulin (α_2_-M) is particularly abundant. It is worth noting that a specific complex between α_2_-M and β_2_-m also circulates in the plasma of hemodialysis patients (13). In 2012, the first natural variant of β_2_-m was discovered in a French family where all the heterozygous carriers of the mutation presented a multi-visceral amyloid deposit (14). Liver, kidney, and heart were all involved, but unexpectedly, bones and ligaments were not affected. This finding was quite surprising in terms of the known tropism of the WT β_2_-m for the muscle-skeletal system. Another unexpected finding was the absence of WT β_2_-m in the deposits, although its intrinsic amyloidogenic propensity is well established. Equally surprising was the absence of N-terminal truncated species, which are ubiquitous constituents of β_2_-m amyloid deposits in dialysis-related amyloidosis (DRA).

In the last two decades, the molecular characterization of amyloid deposits caused by WT β_2_-m in patients under hemodialysis, and more recently the molecular and pathological features of the familial form of β_2_-m, have stimulated seminal studies on the molecular basis of the amyloidogenesis of globular proteins *in vivo*, moving from totally artificial conditions to more bio-compatible methods (Table 1). These studies have provided new insights on the molecular basis of the intrinsic predisposition to amyloid conversion and on the identification of the physical-chemical conditions suitable *in vivo* to prime the conformational transition, as well as some clues on the mechanism responsible for the selective tissue targeting of amyloid deposits in systemic amyloidosis.

**TABLE 1:** **Summary of the different methods reported in literature to generate β_2_-m amyloid fibrils *in vitro***

Experimental conditions	Reference
50 mm sodium citrate, pH 2.5–4, 37 °C	16
100 μm β_2_-m in the presence of seeds	
50 mm sodium citrate, 100 mm NaCl, pH 2.5, 37 °C	58
100 μm β_2_-m	
50 mm sodium citrate, pH 6.5, 37 °C	25
100 μm ΔN6β_2_-m in the presence of seeds	
50 mm sodium citrate, pH 7.3, 37 °C	35
100 μm refolding intermediate in the presence of seeds	
50 mm sodium phosphate, 100 mm NaCl, pH 7.4, 0.5% SDS, 37 °C	19
25 μm β_2_-m in the presence of seeds	
50 mm sodium phosphate, 100 mm NaCl, pH 7.4, 20% TFE,*^a^* 37 °C	20
100 μm β_2_-m in the presence of heparin-stabilized seeds	
25 mm sodium phosphate, pH7.0, 37 °C, stirring at 250 rpm	28
40 μm β_2_-m in the presence of heparin, SAP,*^b^* apolipoprotein E-stabilized seeds	
50 mm ammonium acetate, pH 6.4, 20 μm heparin, fibrillar collagen type I, 37–40 °C	31, 32
40–50 μm β_2_-m	
1 m NaCl, pH 7.5, 37 °C, 24 h stirring, incubation without agitation for 25−45 days	59
30–60 μm β_2_-m	
1 m NaCl, pH 7.5, 60–70 °C, 24 h stirring	60
40–80 μm β_2_-m	
25 mm sodium phosphate, pH 7.4, 37 °C, stirring at 1500 rpm	40
40 μm D76N β_2_-m	

*^a^* TFE, trifluoroethanol.

*^b^* SAP, serum amyloid P component.

## β_2_-m Fibrillogenesis *in Vitro*

The first successful attempt to obtain the fibrillar conversion of native β_2_-m was achieved by Connors *et al.* (15) immediately after the identification of β_2_-m as the causative protein of DRA. This first method was based on the minimization of ion strength and on the maximal increase of β_2_-m concentration. Although the yield was quite low, the study provided the first demonstration that globular β_2_-m can be converted into fibrils *in vitro* and that the concentration represents a crucial condition. A more efficient method of β_2_-m fibrillogenesis was introduced in 1997 by Naiki *et al.* (16). In this case, the massive conversion of β_2_-m into fibrils was primed by the presence of seeds of natural fibrils and required a very low pH. This method highlighted how fibrillogenesis is accelerated by the presence of fibrillar nuclei and in general how the amyloidogenesis requires both a nucleation phase and an elongation phase. Moreover, the low pH implied that the fibrillogenesis of the wild type required the protein unfolding and was perfectly consistent with similar evidences obtained in the same historical phase with other globular amyloidogenic proteins, such as the lysozyme and TTR variants (17, 18). Other ways to unfold the β_2_-m were pursued by the Naiki group (19, 20) using SDS or trifluoroethanol as mild protein denaturants. The possibility to transform native β_2_-m into fibrils led to studies aiming to monitor major conformational changes occurring during the transition and to identify which part of the native molecule is substantially unaltered within the fibrils.

The work carried out by the Goto (21) and Radford (22) teams revealed that low pH fibrillogenesis implies massive conformational changes of the N-terminal and C-terminal portions of β_2_-m. However, the central core of the native protein constrained through its single disulfide bridge is apparently substantially conserved in the fibrils. These data were consistent with the fact that the disulfide bridge bond (residues 25–80) is necessary for the formation of fibrils *in vitro* and that when this is reduced, the β_2_-m can only form amorphous aggregates (23). Moreover, the disulfide bridge is present in natural amyloid fibrils (24). The maintenance of the disulfide bond present in the native protein precursors is common to other types of amyloidosis, and it is particularly meaningful for lysozyme fibrils, where the four disulfide bonds of the native enzyme can be found in the natural fibrils (17).

The structure of the natural fibrillar proteins is very informative and represents a profitable guide for designing proper conditions of fibrillogenesis *in vitro*. This proposition was particularly true in relation to the evidence that truncated species of β_2_-m were ubiquitously found in all the natural amyloid deposits associated with DRA. The removal of six residues at the N terminus of β_2_-m (ΔN6β_2_-m) has a massive impact on the structure, on the stability, and on the fibrillogenic propensity of β_2_-m (25). ΔN6β_2_-m rapidly forms fibrils in physiologic conditions that are otherwise not permissive for full-length WT β_2_-m. This most likely plays a pivotal role in the pathogenesis of the disease, and it can be considered a natural metastable conformer suitable for the nucleation of β_2_-m fibrils. ΔN6β_2_-m rapidly forms oligomers in solution even at very low concentrations, and it catalyzes the oligomerization of WT β_2_-m even in a physiologic environment (26). The Radford group (27) has extensively investigated the structural features of ΔN6β_2_-m and its capacity to recruit the WT into amyloid fibrils. The NMR spectra revealed a remarkable overlapping of the structure of ΔN6β_2_-m with the structure of the P32G variant that was prepared to investigate the effect of the transition *cis-trans* of Pro-32 on β_2_-m fibrillogenesis. When Pro-32 is in *cis* conformation, β_2_-m is protected from self-aggregation, whereas the *trans* conformation makes the β_2_-m highly susceptible to a rapid fibrillar conversion (28, 29).

The role of selective proteolytic cleavage in amyloidogenesis has been extensively debated. However, there are still doubts on the timeline of the proteolytic cleavage, notably if the event precedes the fibrillar aggregation or if it simply represents a process of trimming of the less protected portion of the polypeptide cemented in the amyloid fibrils. Unequivocal demonstration of a pre-fibrillogenic proteolytic event is still missing; however, it is unquestionable that selective proteolytic cleavage mimicking that occurring *in vivo* has a strong enhancing effect on the kinetics of fibril formation of β_2_-m and other proteins such as TTR (30). Despite the relevance of proteolytic cleavage, some types of amyloid fibrils, such as those formed *in vivo* by variants of lysozyme (17) and the natural variant of β_2_-m D76N (14), only contain an intact and full-length mature protein.

**FIGURE 1.**
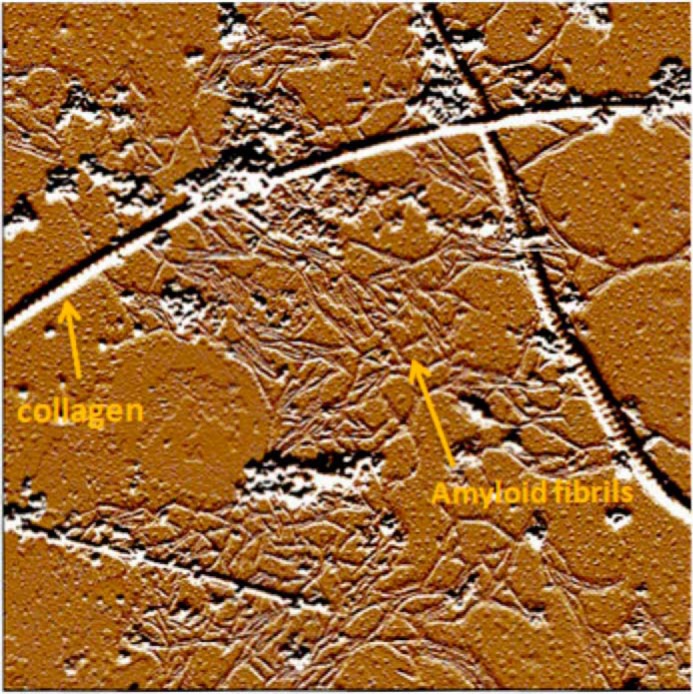
**Tapping mode atomic force microscopy image of β_2_-m amyloid fibers in the presence of fibrillar collagen and heparin.** Shown is the fibril network connecting isolated collagen fibrils, observed after 24 h of incubation. Non-fibrillar aggregates are also present. Amplitude data: scan size, 5.7 μm. This figure was originally published in *The Journal of Biological Chemistry* (Relini, A., De Stefano, S., Torrassa, S., Cavalleri, O., Rolandi, R., Gliozzi, A., Giorgetti, S., Raimondi, S., Marchese, L., Verga, L., Rossi, A., Stoppini, M., and Bellotti, V. (2008) Heparin strongly enhances the formation of β_2_-microglobulin amyloid fibrils in the presence of type I collagen. *J. Biol. Chem.* **283**, 4912–4920. © the American Society for Biochemistry and Molecular Biology).

The discovery that *in vitro* truncated β_2_-m can form amyloid fibrils, in a physiologic environment, has moved the methods of *in vitro* fibrillogenesis toward more bio-compatible conditions. A successful example of a bridge between the known tropism of β_2_-m for the muscle skeletal system and an *in vitro* method was achieved by studying the effect of type I and type II collagen on β_2_-m amyloidogenesis (31). The effect of the interaction of β_2_-m with the collagen's surface is remarkable, and Fig. 1 illustrates a representative image of the growth of amyloid fibrils stemming from type I collagen fibers. The presence of β_2_-m oligomers and GAGs was able to accelerate the process of the amyloid grown on the collagen surface. This confirmed the generic pro-amyloidogenic effect played by the aforementioned components in fibrillogenesis (32). However, despite the growth of fibrils on the collagen surface, in the absence of fluid flow, the majority of the bulk of WT β_2_-m in solution was not converted into fibrils on a time scale of several days.

**FIGURE 2.**
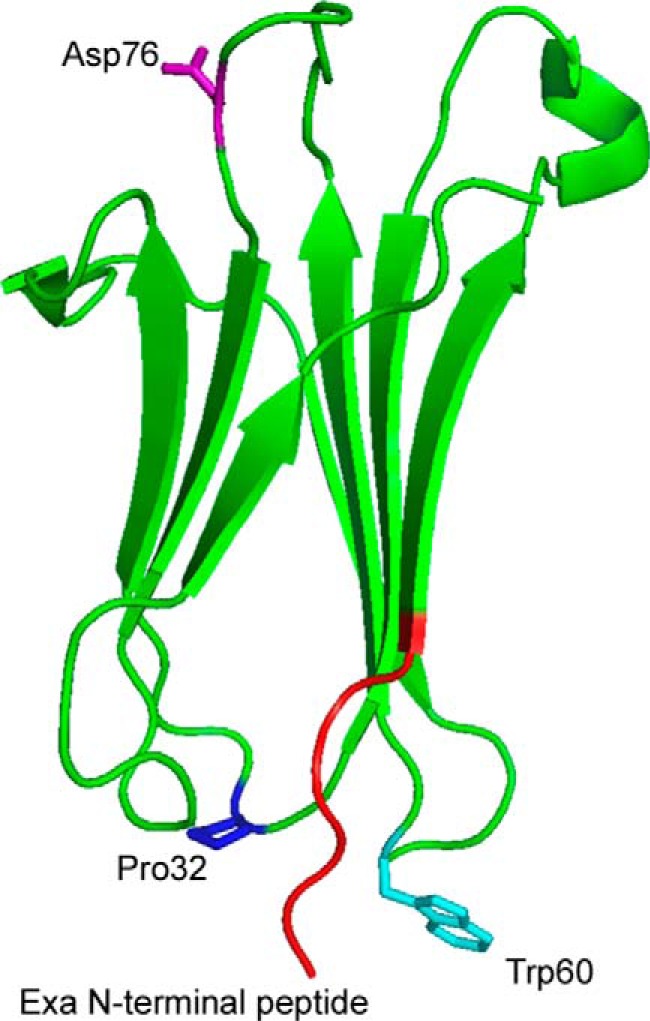
**β_2_-m structure (Protein Data Bank (PDB) entry 2yxf) highlighting the N-terminal peptide and three key residues: Pro-32, Trp-60, and Asp-76.** *Exa N-terminal peptide*, the six N-terminal residues lacking in ΔN6β_2_-m.

β_2_-m, similar to many other globular amyloidogenic proteins, displays a strong propensity to spontaneously aggregate into soluble oligomers; in particular, β_2_-m does so in the physiologic buffer (26, 28, 33). It is likely that the oligomerization is mainly primed by a small population of partially folded conformers in equilibrium with the fully folded protein. NMR studies, molecular dynamic simulation, capillary electrophoresis, and spectroscopic analysis highlighted the existence of a partially folded intermediate, initially named I2 (34, 35), in which specific regions of the molecule exhibit a very high flexibility and rapid structural fluctuations (36). The fluctuation toward a partially folded state is probably dictated by the peculiar dynamics of the loop interconnecting the strands D and E. This loop is particularly unstable, most likely due to the presence of a tryptophan (Trp-60) in the center of the loop (37, 38) (Fig. 2).

Remarkably, despite an unfavorable thermodynamic effect, a Trp is present in this position in almost all the vertebrate species (39), and it is crucial for the proper binding of β_2_-m to the heavy chain of the major histocompatibility complex class I (MHCI). In human β_2_-m, the Trp-60 can therefore play two contrasting roles: a functional physiologic contribution to the correct assembly of the major histocompatibility complex class I and a detrimental, destabilizing, and pro-amyloidogenic effect when the plasma concentration of β_2_-m is persistently high.

## What Did the Comparison between the Amyloidogenesis Mechanism of the Wild Type and the Rare D76N Variant Reveal?

Elucidation of the molecular mechanisms of rare diseases can promote crucial progress in the interpretation of more general phenomena as well as illuminating the mechanisms of common diseases. The discovery of the rare natural amyloidogenic variant of β_2_-m allowed a systematic analysis of its pathological and biochemical features as well as providing the opportunity to correlate specific molecular characteristics of the WT and of the variant with peculiar clinical presentations and different pathological features of the two related types of amyloidosis. The first, associated with hemodialysis, is caused by the persistently high concentration of β_2_-m in plasma, and it is characterized by a selective localization of amyloid in bones and ligaments. The genetic form is not associated with any increase of β_2_-m in plasma, and the amyloid localization is mainly visceral, involving liver, spleen, kidney, and heart, whereas, quite surprisingly, bones and ligaments are spared. The original question was: as far as this variant is concerned, which theories, hypothesized to explain amyloidogenesis of WT β_2_-m, do remain applicable? This question became particularly cogent once we discovered the destabilizing effect of the mutation and the possibility to convert the D76N variant into amyloid fibrils in physiologic buffer, by simply exposing the protein to fluid shear forces in the presence of natural or artificial hydrophobic surfaces (40).

In WT β_2_-m, its intrinsic amyloidogenic propensity was ascribed to its dynamic properties, particularly evident in the D strand and in the D-E loop region, whose flexibility is propelled by the above mentioned Trp-60 (38). The comparative measurement of the folding and unfolding kinetics as well as of the native protein's unfolding free energy allowed us to establish that ∼5% of WT β_2_-m physiologically populates a partially folded intermediate state and that the mutation D76N causes a 5-fold enhancement of the concentration of the intermediate at the equilibrium in the physiological buffer (40).

Therefore, a direct correlation exists between the amyloidogenic propensity of β_2_-m species and the concentration of this partially structured intermediate. The structure of this intermediate was extensively investigated, but it is still elusive due to the high conformational dynamic on a microsecond to millisecond time scale. A brilliant approach to single out specific characteristics of this intermediate is based on the discovery that several structural and functional features overlap those of ΔN6β_2_-m. A remarkable feature of both ΔN6β_2_-m and the folding intermediate I2 is most certainly the *trans* conformation of the His-Pro-32 peptide bond (27). The *cis-trans* transition is crucial for the fibrillogenesis, and it is a hallmark of the fibrillar state (41, 42).

Although the amyloidogenic potential of the two β_2_-m species (WT and D76N) is in agreement with the concentration of the partially folded state at the equilibrium, a major and peculiar difference emerges from the clinical/pathological features of the two types of amyloidoses: notably, the localization in bones and ligaments of the WT and the visceral localization of the D76N. The amyloid deposition in bones is quite rare in other systemic amyloidoses, but it is an unavoidable complication of DRA. The specific localization in bones and ligaments of the amyloidosis caused by hemodialysis was ascribed to a peculiar affinity of β_2_-m for type I and type II collagen. Nonetheless, the measurement of the affinity constant of β_2_-m for collagen revealed a weak *K_d_* of >1 × 10^−4^ m (43). It is plausible to believe that preferential accumulation of β_2_-m in collagen becomes significant only for the micromolar concentration during hemodialysis, whereas it does not become so in physiological sub-micromolar concentrations. Furthermore, it is likely that in DRA, the truncated ΔN6β_2_-m species, which has a 10-fold higher affinity to the full-length WT for collagen (43), is a potent promoter of amyloidogenesis on the collagen surface and that this is present only in the amyloid deposits localized in bones and ligaments. The latter suggests that a protease, well represented in the aforementioned tissues, could cleave β_2_-m in its first strand, consequentially accelerating the local accumulation of this conformer, which is then rapidly followed by its fibrillar aggregation.

The mechanism by which collagen facilitates the amyloidogenesis of β_2_-m is uncertain. Collagen offers wide hydrophobic surfaces, and it is known that the flow of a physiologic fluid, at the interface between polar and hydrophobic surfaces, can generate sufficient forces to partially or totally unfold a globular protein (44). Hydrodynamic shear stress alone can generate forces that can be quantified through the equation

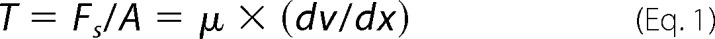
 where *T* represents the shear stress, *F_s_* the shear force, *A* is the cross-sectional area of the molecule, μ is the dynamic viscosity of the fluid, and *dv/dx* is the shear rate, which is the fluid velocity gradient.

However, the hydrophobic forces (*F*_Hydro_) acting on the molecule in the extracellular space play a dominant role in the protein destabilization, and they can be calculated according to Mangione *et al.* (40) through the equation.

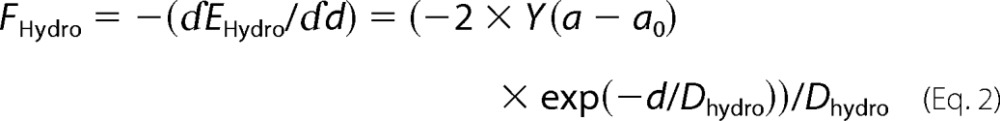
 where *E*_Hydro_ represents the hydrophobic interaction energies between two apolar surfaces, *Y* is the interfacial tension, *d* is the distance between the two surfaces, *a* is the exposed area of the molecule at distance *d*, *a*_0_ is the optimum exposed area of the molecule, which we consider to be equal to the area of one amino acid, and *D*_hydro_ is the hydrophobic decay length.

**FIGURE 3.**
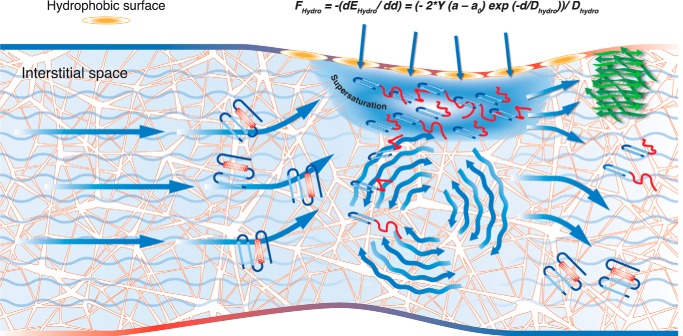
**Schematic picture of the hypothetical events occurring in the interstitial space where globular soluble proteins undergo fibrillar conversion.** The chemical physical characteristics of the interstitial space and forces generated by the fluid flow are well reviewed by Swartz and Fleury (61). Native globular proteins flow through a network of fibrous proteins (*i.e.* collagen and elastin) and GAGs. These matrix proteins expose hydrophobic patches with which the native globular proteins collide. At the interface between the hydrophobic surface and the aqueous fluid, proteins are exposed to forces sufficient to perturb the folded state. The exposure of normally buried hydrophobic elements further facilitates the interaction with the hydrophobic matrix, local accumulation of partially folded globular conformers reaching a condition of supersaturation. Supersaturation is the precondition for protein aggregation and loss of solubility. Even minimal changes in the intensity of the shear flow can break the very labile soluble state of partially folded proteins when they reach the condition of supersaturation. If supersaturation is not reached, the simple unfolding of the proteins does not imply a fibrillar conversion and the protein can properly refold and escape from the aggregation.

We hypothesize that, in the extracellular matrix in the very thin fluid space at the interface between hydrophobic surfaces and the interstitial fluid, the amyloidogenic proteins could partially unfold and expose normally buried hydrophobic patches. These partially folded conformers could locally accumulate and reach a condition of supersaturation (Fig. 3). Such a state is extremely unstable, and several events can break solubility, leading to protein precipitation. Although the shear flow of the fluid is not *per se* sufficient to unfold globular proteins, it may play a fundamental role in breaking the condition of supersaturation. In fact, in conditions of supersaturation, the intensity of shear flow inversely correlates to the lag phase of β_2_-m fibrillogenesis (45). All these data suggest that the concentration of β_2_-m and its level of thermodynamic stability could direct the amyloid formation in two distinct tissue targets. In conditions of high concentration, but relatively good thermodynamic stability, the amyloid is deposited in bones and ligament. When plasma concentration is low, implying a negligible accumulation of β_2_-m on the collagen surface, bones and ligaments are spared. If a mutation reduces the stability of β_2_-m (*i.e.* D76N mutation), the shear stress in the extracellular matrix of visceral organs such as liver, spleen, kidney, and heart is sufficient to unfold the unstable variant and prime a cascade of events as represented in Fig. 3. It is worth noting that the amyloid deposits of patients heterozygous for the mutation D76N do not contain the WT β_2_-m. However, *in vitro*, once D76N fibrils are formed, shear stress, generated by the dynamics of a physiologic fluid and the exposure to hydrophobic surface of biological molecules, can also prime amyloidogenesis of WT β_2_-m (40). These findings are apparently incompatible, but let us grasp the complexity of amyloidogenesis *in vivo*. The demonstration that, in the presence of a generic extracellular chaperone such as αB crystallin, even in a very low molar ratio, the WT β_2_-m becomes resistant to the fibrillar conversion induced by the D76N fibrils (40) suggests that *in vivo* factors such as chaperones can modulate the amyloidogenesis of WT proteins.

## The Existence of Natural Factors Modulating Amyloidogenesis

The discovery that forces physiologically harboring the human tissues are sufficient to prime the protein unfolding and fibrillogenesis (44) sheds light on the pathophysiology of amyloid formation. However, a deep gap still exists between the *in vitro* and *in vivo* conditions of protein amyloidogenesis. The *in vitro* investigation usually concerns the transition of an isolated and homogeneous molecule, but *in vivo* the amyloidogenic protein interplays with a variety of other chemical entities and peculiar physical environments. *In vivo* an equilibrium exists between pro-fibrillogenic and anti-fibrillogenic factors. In the interstitial space, where the amyloid deposits are formed, a few proteins could specifically influence the aggregation propensity of amyloidogenic proteins. Albumin *per se*, which is present in the interstitial space at a concentration around 10 mg/ml, can partially inhibit the fibrillogenesis by improving the protein's colloidal stability. However, more specific and more effective inhibitors of protein aggregation are present in the extracellular space where chaperones play a rather important role (46). In β_2_-m amyloidosis, the anti-fibrillogenic properties of some of these chaperones were specifically studied, namely the α_2_-M (47) and, as mentioned above, αB-crystallin (48). The mechanism by which *in vitro* α_2_-M interferes with β_2_-m amyloidogenesis reveals that the most effective species is a dimeric form of α_2_-M, resulting from the stress-mediated dissociation of the tetramer. It was demonstrated that α_2_-M binds the unfolded β_2_-m more avidly than the folded one (47). These findings suggest that conditions promoting at once the unfolding of the amyloidogenic protein as well as the structural rearrangement of the chaperone could activate the chaperone function of α_2_-M. Consistent with the *in vitro* and *ex vivo* studies, it was also demonstrated that although α_2_-M inhibits β_2_-m aggregation, it is nonetheless unable to disaggregate mature fibrils. Other extracellular chaperones display similar properties. αB-crystallin is capable of inhibiting fibrillogenesis when added in sufficient quantity to compete with the self-assembly of β_2_-m variant and, as mentioned above, to dissociate the aggregation of WT and variant (48). However, once the fibrils are formed, chaperones, such as α_2_-M, are unable to solubilize the amyloid and co-precipitate with the mature fibrils.^3^

The general function of extracellular chaperones in amyloidogenesis is well characterized in clusterin. The capacity of clusterin to interfere with amyloid formation was not tested on β_2_-m, but on apolipoprotein C-II (49), lysozyme (50), synuclein, and other amyloidogenic proteins (51, 52). Similarly to α_2_-M and crystallin, clusterin is also unable to dissociate preformed fibrils, and it is frequently found as a bystander component of natural amyloid fibrils.

The emerging scenario is consistent with the hypothesis that these extracellular chaperones could perform a dual, apparently antithetical function, notably the inhibition of oligomerization and fibrillogenesis acting on the solubility of partially folded intermediates as well as the stabilization of amyloid fibrils once these are formed. The balance between these two functions in the formation of amyloid in the natural environment is yet to be determined, but it is most probably crucial for the elucidation of the natural history of the disease and the possible therapeutic exploitation of these molecules.

## β_2_-m Pharmaceutical Interactants and Amyloidogenesis Inhibition

Besides extracellular chaperone proteins, other compounds can inhibit β_2_-m amyloidogenesis through different mechanisms. As a prototype of a generic inhibitor of amyloidogenesis, we proved that *in vitro* the antibiotic doxycycline can inhibit the amyloidogenesis of WT β_2_-m (53), and with a similar dose, it can affect the fibrillogenesis of the natural variant D76N.^3^ The main obstacle to *in vivo* doxycycline therapeutic efficacy was expected to be the difficulty in obtaining a therapeutic concentration. However, the concentration of doxycycline in the tissue targets proved to be much higher than in plasma (54) and most likely sufficient to inhibit aggregation of oligomers and β_2_-m toxicity. Preliminary data, obtained in the first three patients affected by DRA and treated with doxycycline, suggest that a therapeutic response can be achieved even with a plasmatic concentration that is apparently insufficient to abrogate fibrillogenesis *in vitro* (55). Specific small ligands of an amyloidogenic protein can be therapeutically used, as the ligand-mediated stabilization can be sufficient to protect from the unfolding and aggregation. The best example for this approach is the stabilization of TTR through small analogues of its natural ligands (56). However, no specific small ligands with similar properties are available for β_2_-m. An immunological approach, mainly based on the use of specific antibodies, can probably be properly pursued for this amyloidosis. We previously showed that the specific monovalent single chain camelid antibodies can inhibit wild type amyloidogenesis (57), and we are confirming their effect on the Asn-76 variant.^3^ In the absence of other possible therapies and considering the relatively low concentration of circulating β_2_-m, a treatment-based antibody should be pursued in the familial form of β_2_-m amyloidosis, and once its efficacy is demonstrated, it could be extended to other forms of systemic amyloidoses.

## Concluding Remarks

The extensive research carried out on β_2_-m-related amyloidosis has substantially contributed to elucidating the general rules dictating the amyloid conversion of globular proteins in systemic amyloidosis. To self-aggregate into a cross-β structured fibrils, the amyloidogenic globular protein must partially unfolded. The loss of the native structure can be primarily caused by mutations of covalent modifications such as limited proteolytic cleavages. WT β_2_-m is a paradigmatic example of how the aggregation risk of some proteins, exposing hydrophobic and flexible regions for functional reasons, is controlled by maintaining very low protein concentrations. The risk of protein aggregation becomes instead extremely high when a condition of supersaturation of the partially folded intermediates occurs in the extracellular space of the target organs. Investigations of β_2_-m models have provided unique new insights elucidating the mechanism of selective tissue targeting in other forms of systemic amyloidosis, where the amyloid is deposited in organs distant from the synthesis site. The most common forms of systemic amyloidosis, such as those caused by immunoglobulin light chains, TTR, lysozyme, and lipoproteins, are prototypic examples of instances when the sites of production and of major deposition are totally different. It is now possible to address the crucial and challenging question of the role played by local tissue factors in favoring and contrasting protein aggregation. The equilibrium between these factors probably determines the peculiar natural history of the different types of systemic amyloidosis. It is plausible that even within the same type of amyloid diseases, these factors will influence the precise medical and pathologic features of the individual patient.

## Footnotes

3 V. Bellotti *et al.*, unpublished data.

## Footnotes

2 The abbreviations used are:

β_2_-m: β_2_-microglobulin
ΔN6β_2_m: truncated β_2_-m isoform lacking the 6 N-terminal residues
α_2_-M: α_2_-macroglobulin
DRA: dialysis-related amyloidosis
TTR: transthyretin
GAG: glycosaminoglycan.
